# Predictive factors for outcomes of sialoendoscopy

**DOI:** 10.1016/j.bjorl.2025.101631

**Published:** 2025-05-14

**Authors:** Hannah Damasceno Barreto, Jose Higino Steck, Samuel Serpa Steck, Diogo Silva de Carvalho, Carlos Takahiro Chone

**Affiliations:** a Universidade Estadual de Campinas (UNICAMP), Departamento de Otorrinolaringologia e Cirurgia de Cabeça e Pescoço, São Paulo, SP, Brazil; b Universidade Estadual Paulista de Botucatu (UNESP), Botucatu, SP, Brazil; c Pontifícia Universidade Católica de Campinas (PUC), Campinas, SP, Brazil

**Keywords:** Sialoendoscopy, Sialadenitis, Sialolithiasis

## Abstract

**Objectives:**

We evaluated the epidemiological and clinical factors related to the complications and failure of sialoendoscopy.

**Methods:**

This retrospective study analyzed the medical records of patients who underwent sialoendoscopy between 2010 and 2019.

**Results:**

Of the 174 sialoendoscopies analyzed, 125 (71.8%) were performed in women, and 114 (65.5%), in the parotid gland. The most prevalent complications described were false path (4.59%) and restenosis (2.30%). Complications in the submandibular gland were 3.43 times more common than those in the parotid gland and procedures in that gland were less successful (86.7% vs. 91.2%). Sialolithiasis treatment was most strongly associated with complications and failure then the other groups.

**Conclusion:**

Sialoendoscopy has uncommon and limited complications. Procedures in the submandibular gland and for sialolithiasis treatment were more likely to result in complications and recurrences.

**Level of evidence:**

4.

## Highlights

• Chronic sialoadenitis is mainly treated with surgical procedures.
• Sialoadenectomies may result in aesthetic defects and complications.
• Sialoendoscopy is an effective treatment for major salivary gland duct diseases.
• Sialoendoscopy boasts minimal complications and high success rates.

## Introduction

Acute salivary gland diseases have an incidence of 0.173 cases per 10,000 people and most commonly affects men aged 50–70 years old. The etiologic diagnose and treatment could involve surgery or sialoendoscopy when symptoms are recurrent.^1^

Endoscopic salivary gland surgery (sialoendoscopy) was introduced in France between 1990 and 2000. In Germany, it began to emerge in 2000 by Katz and was later improved by Gundlach. This technique is used to diagnose and treat Chronic Obstructive Salivary Gland Diseases (COSGD), as sialolithiasis, autoimmune disorders, juvenile recurrent parotitis, and radioiodine therapy-induced sialadenitis. The rate of clinical improvement is 76%–100%.The gland preservation rate is 88%–91% in literature.^2^, ^3^, ^4^

There were few studies that analyzed the predictive factors of sialoendoscopy for clinical improvement, recurrence of and complications of procedure in a large sample size of patients with Chronic Obstructive Salivary Gland Diseases (COSGD). The finding of these factors could improve the clinical practice of sialoendoscopy helping surgeons in decision making.

The objective of our study was to evaluate the predictive factors for clinical improvement, recurrence, and complications of sialoendoscopy in a large retrospective cohort of consecutive COSGD patients with multivariate analysis.

## Methods

We evaluated, retrospectively, consecutive patients with COSGD who underwent sialoendoscopy between September 2010 and November 2019 by a single surgeon.

The patients indicated for the procedure had frequent swelling with pain recurrent that has not responded to the clinical conservative treatment. All patients were examined by ultrasonography of the salivary glands and/or computerized tomography in order to exclude neoplastic diseases and evaluate inflammatory findings in ductal system as dilation, stenosis or stones. The contraindication of the procedure was acute suppurative sialadenitis or patients with severe trismus.

These procedures were performed under total venous anesthesia. The surgical instruments were dilators, guide wires, scopes with two or three working channels, a 0.6 mm semi-flexible sialoendoscopic optic fiber (Karl Storz®), baskets, balloons, and biopsy forceps (Karl Storz®). The parotid or submandibular gland ostia were visualized and progressively dilated (Schaitkin Salivary Dilators; Karl Storz®). If ostia were unable to be dilated due to severe obstruction, an incision (cut down) was made to find the distal portion of the duct (closest to the ostia). A sialendoscope (Karl Storz®) was used to navigate each salivary duct and through the working channels the cause of obstruction was treated using the instruments.

The analyzed clinical parameters were age, sex, etiology, sialoendoscopy description, intraoperative and postoperative complications, and outcomes.

Therapeutic failure was defined as the need for a second sialoendoscopy within a period of <6-months from the first one, according to previous studies.^4^, ^5^, ^6^ Clinical improvement was assessed using the COSS – Chronic Obstructive Sialadenitis Symptoms questionnaire. This questionary is an instrument to assist in the analysis of sialadenitis symptom severity. COSS scores under 10 correlates with a significant improvement in symptoms, whereas scores between 10 and 25 correlate with partial resolution. The score ranges from 0 to100 and includes 20 topics: pain severity, pain frequency, xerostomia, noticeability by others, level of embarrassment, swelling, purulent exsudate, interference with daily activities, among others.^6^, ^7^

Our outcomes were procedure recurrence, complications, sialoendoscopy findings and clinical improvement. Those are compared to demographic and clinical parameters to find predictors of our outcomes. Patients were grouped according to their etiology for sialadenitis.

Patients whose sialoendoscopy fibers did not go through the duct or without surgical description of finding were excluded from the analysis. We excluded from the study patients with previous procedures in salivary glands, radiation therapy or neoplastic disease in salivary glands.

### Statistical analysis

Summary measures of means, standard deviations, frequencies and percentages were investigated with statistical analysis with R version 4.3.0. Copyright (C) 2023 The R Foundation for Statistical Computing. Continuous dependent variables had a non-normal distribution. Continuous outcomes were studied with Kruskal-Wallis and Mann-Whitney tests. For categorical data we used Chi-Square test.

## Results

We analyzed 174 procedures, patients aged 3–89 years, which were divided into five different groups according to their etiology.

Sialolithiasis, post-radioiodine therapy, autoimmune disease and non-specific chronic sialadenitis are evaluated in Table 1 with a total of 160 procedures. The non-specific chronic sialadenitis group included patients with the highest mean age (57.4 years), and the post-radioiodine therapy group included the youngest patients (38.9 years) and largest proportion of women (92.9%).

**Table 1:** Demographic and clinical variables according to etiology of salivary gland disease with statistical non-parametrical evaluation (Kruskal–Wallis or Chi-Square test) n = 160.

Variable	Sialolithiasis (n = 51)	Post-radioiodine therapy (n = 42)	Autoimmune disease (n = 34)	Non-specific chronic sialadenitis (n = 33)	*p-*valor
Age: years (SD)	51.6 (16.8)	44.6 (14.6)	41.6 (16.5)	57.7 (17.1)	**<0.001**
Follow-up: months (SD)	14.0 (20.7)	27.6 (29.4)	9.06 (13.9)	16.4 (22.5)	**0.003**
Recurrence within 6 months (%):					**0.017**
No	43 (84.3%)	42 (100%)	29 (85.3%)	31 (93.9%)	
Yes	8 (15.7%)	0 (0.00%)	5 (14.7%)	2 (6.06%)	
Gender (%):					**<0.001**
Female (%)	27 (52.9%)	39 (92.9%)	30 (88.2%)	20 (60.6%)	
Male (%)	24 (47.1%)	3 (7.14%)	4 (11.8%)	13 (39.4%)	
Affected gland (%):					**<0.001**
Parotid	19 (37.3%)	37 (88.1%)	32 (94.1%)	14 (42.4%)	
Submandibular	32 (62.7%)	5 (11.9%)	2 (5.88%)	19 (57.6%)	
Side (%):					0.575
Right	29 (56.9%)	19 (45.2%)	15 (44.1%)	15 (45.5%)	
Left	22 (43.1%)	23 (54.8%)	19 (55.9%)	18 (54.5%)	
Complications:					**0.014**
No	41 (80.4%)	41 (97.6%)	33 (97.1%)	28 (84.8%)	
Yes	10 (19.6%)	1 (2.38%)	1 (2.94%)	5 (15.2%)	

Results are expressed as mean (standard deviation) or n (%).

Juvenile recurrent parotitis was analyzed apart because of the different age-specific commitment of the child population (<18-years), in Table 2 with 14 patients.

**Table 2:** Profile of patients in the juvenile recurrent parotiditis.

Variable	Juvenile recurrent parotitis (n = 14)
Age: years (SD)	8.79 (5.42)
Follow-up: months (SD)	11.8 (17.1)
Relapse within 6-months:	
No (%)	11 (78.6%)
Yes (%)	3 (21.4%)
Sex:	
Female (%)	9 (64.3%)
Male (%)	5 (35.7%)
Affected gland:	
Parotid (%)	12 (85.7%)
Submandibular (%)	2 (14.3%)
Side:	
Right (%)	4 (28.6%)
Left (%)	10 (71.4%)
Complications:	
No	13 (92.9%)
Yes	1 (7.14%)

Results are expressed as mean (standard deviation) or n (%).

Parotid gland was more affected in post-radioiodine therapy and autoimmune diseases although submandibular gland in sialolithiasis and chronic sialadenitis patients (Table 1).

The sialoendoscopy findings were described in Table 3. Among these 174procedures, there were 18 cases of recurrence (Table 4) and 18 events considered complications (Table 5). The comparison of recurrences or complications with sialendoscopy findings did not show us statistically significant differences (*p* > 0.05).

**Table 3:** Frequency of sialoendoscopy findings by etiology.

Group	Procedures	Finding	Nº of events	Frequency of occurrence
Sialolithiasis		Stone	43	84%
	51	Stenosis	17	33%
		Mucus plug	3	6%
		Pallor	11	2%
Post-radioiodine therapy		None	1	2%
		Stenosis	31	74%
	42	Mucus plug	13	31%
		Pallor	13	31%
Juvenile recurrent parotitis		Stenosis	11	79%
	14	Pallor	5	36%
		Mucus plug	2	14%
Autoimmune disease		Stenosis	20	59%
		Pallor	18	53%
	34	Mucus plug	11	32%
		None	1	3%
Non-specific chronic sialadenitis		Stenosis	24	73%
	33	Pallor	6	18%
		None	6	18%
		Mucus plug	3	9%
Total	174	Stenosis	92	44%
		Pallor	38	18%
		Stone	43	20%
		Mucus plug	30	14%
		None	8	4%

**Table 4:** Comparison between patients with and without recurrence relating to sialoendoscopy findings (Chi-Square test).

Finding	Recurrence		*p*-value
	No (n = 156)	Yes (n = 18)	
Stone:			0.155
No	120 (76.9%)	11 (61.1%)	
Yes	36 (23.1%)	7 (38.9%)	
Mucus plug:			0.332
No	129 (82.7%)	13 (72.2%)	
Yes	27 (17.3%)	5 (27.8%)	
Stenosis:			1.000
No	64 (41.0%)	7 (38.9%)	
Yes	92 (59.0%)	11 (61.1%)	
Pallor:			1.000
No	117 (75.0%)	14 (77.8%)	
Yes	39 (25.0%)	4 (22.2%)	
None:			1.000
No	148 (94.9%)	18 (100%)	
Yes	8 (5.13%)	0 (0.00%)

**Table 5:** Comparison between patients with and without complication relating to sialoendoscopy findings (Chi-Square test).

Finding	Complication		*p*-value
	No (n = 156)	Yes (n = 18)	
Stone:			0.155
No	120 (76.9%)	11 (61.1%)	
Yes	36 (23.1%)	7 (38.9%)	
Plug mucoso:			0.202
No	125 (80.1%)	17 (94.4%)	
Yes	31 (19.9%)	1 (5.56%)	
Stenosis:			1.000
No	64 (41.0%)	7 (38.9%)	
Yes	92 (59.0%)	11 (61.1%)	
Pallor:			1.000
No	117 (75.0%)	14 (77.8%)	
Yes	39 (25.0%)	4 (22.2%)	
None:			0.590
No	149 (95.5%)	17 (94.4%)	
Yes	7 (4.49%)	1 (5.56%)	

The most frequent complication was intraoperative: false path (n = 8, 4.59%). During the postsurgical period, the following events were considered complications: restenosis (n = 4, 2.30%), infection (n = 2, 1.15%), transient lingual paresthesia (n = 2, 1.15%), seroma (n = 1, 0.57%) and salivary fistula (n = 1, 0.57%).

Procedures performed in the submandibular gland duct were more frequently associated with complications (*p* = 0.025). The complication rate was 18.33% (11/60 cases) in the submandibular procedures and 6.14% (7/114 cases) in the parotid ones (Table 6). More complications were seen in the sialolithiasis group than in the other groups (*p* = 0.027). No significant differences in age (*p* = 0.215), sex (*p* = 0.811), or side (*p* = 0.314) were observed between groups.

**Table 6:** Comparison between patient profiles and complications (Student's *t*-test or Chi-Square test).

Variable	Complication		*p-*value
	No (n = 156)	Yes (n = 18)	
Age years (SD)	45.0 (19.6)	51.6 (20.7)	0.215
Sex:			0.811
Female (%)	113 (72.4%)	12 (66.7%)	
Male (%)	43 (27.6%)	6 (33.3%)	
Affected gland:			**0.025**
Parotid (%)	107 (68.6%)	7 (38.9%)	
Submandibular (%)	49 (31.4%)	11 (61.1%)	
Side:			0.314
Right (%)	71 (45.5%)	11 (61.1%)	
Left (%)	85 (54.5%)	7 (38.9%)	
Group:			**0.027**
Sialolithiasis (%)	41 (26.3%)	10 (55.6%)	
Post-radioiodine therapy (%)	41 (26.3%)	1 (5.56%)	
Juvenile recurrent parotitis (%)	13 (8.33%)	1 (5.56%)	
Autoimmune disease (%)	28 (17.9%)	5 (27.8%)	
Non-specific chronic sialadenitis (%)	45.0 (19.6)	51.6 (20.7)	0.215

Results are expressed as mean (standard deviation) or n (%).

All the analyzed variables, except diagnosis, were included in a multiple logistic regression model with stepwise variable selection criteria (Table 7). Patients with an affected submandibular gland were 3.43 times more likely to develop complications than those with an affected parotid gland.

**Table 7:** Univariate logistic regression to assess the chance of complication.

Factor	Level	Reference	OR	95% CI (OR)	*p*-value
Age	Continuous	‒	1.02	0.99 – 1.05	0.185
Gender	Male	Female	1.32	0.43 – 3.61	0.607
Affected Gland	Submandibular	Parotid	3.43	1.27 – 9.83	**0.016**
Side	Left	Right	0.53	0.19 – 1.42	0.215

**Fig. 1:**
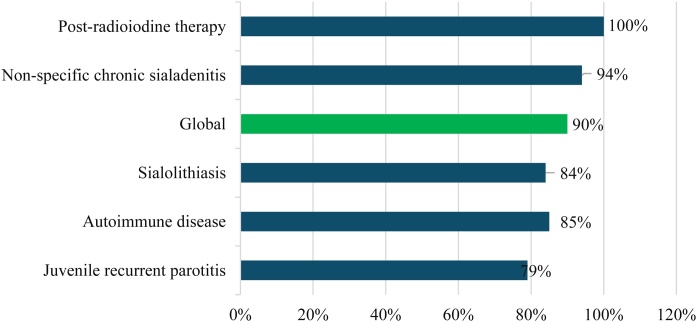
Success rate of sialoendoscopy by groups. Source: Original research results.

The overall success rate of the procedure was 90%. The highest success rate was seen in the post-radioiodine therapy group (100%), and the lowest, in the juvenile recurrent parotitis group (79%) (Fig. 1).

## Discussion

We have seen in our cohort of patients more procedures in women and parotid gland with a frequency of 71.8% and 65.5%, respectively. The differences in frequency between the sexes and glands may be related to the number of sialoendoscopies indicated for the treatment of post-radioiodine therapy sialadenitis and autoimmune diseases (approximately 50% of cases), which are more prevalent among women and in the parotids.^8^, ^9^, ^10^, ^11^, ^12^ As thyroid cancer is more incident and prevalent in women and radioiodine with iodine (I-131) therapy is an adjuvant treatment, it is expected this higher rate among then.^13^ Forty-two patients were related to radioiodine therapy, and 39 (92.9%) of whom were women. Approximately 20% of the used dose is secreted in saliva where its concentration is 20–100 times greater than that in plasma. Furthermore, there is an evident tropism of iodine in glands with a serous acinar cell type, which is more common in the parotid glands.^8^ The clinical improvement rate in this group was 100%. In a meta-analysis, the success rate of sialoendoscopy, specifically for treating post-radioiodine therapy sialadenitis, was 50%–100%; furthermore, the examination findings in these patients and their frequencies were as follows: mucus plug, 47%; stenosis, 40%; and combination of both, 13%. Our findings were as follows: stenosis, 74%; mucus plug, 31%; and pallor of the duct wall, 31%.^8^, ^9^, ^10^, ^14^, ^15^

Autoimmune diseases are also more common in the female population. Sjögren syndrome is frequently associated with recurrent sialadenitis. Thirty-one of 34 cases of sialadenitis were associated with autoimmune diseases and women. The incidence of parotitis is higher in patients with Sjögren's syndrome than in those with submandibular sialadenitis.^6^ Sialoendoscopy in patients with Sjögren's syndrome, complete resolution of the inflammatory obstructive condition was seen in 72% of patients. In this study, the efficacy rate of the procedure in treating autoimmune diseases was 87.2%, and the most frequent sialoendoscopy finding was stenosis (59%), similar to our findings. Pallor (53%) was also a very common finding.^11^

Studies on sialoendoscopy in the pediatric population have reported a success rate of approximately 73%–81% in treating juvenile recurrent parotitis. This was considerably lower than that for procedures performed in adults. A clinical diagnosis of juvenile recurrent parotitis is made when the patient exhibits salivary gland swelling, pain, fever, and at least two annual recurrences. The etiology of the disease is unknown; however, studies have suggested that it is an immune-mediated disease that causes recurrent inflammation of the salivary ducts, particularly the parotid ducts. This inflammation may lead to ductal stenosis and vascular compromise and tends to improve by the end of puberty in most cases. Permanent fibrotic disease is rare. Therefore, it is understood that the inflammatory process persists even after sialoendoscopy. The relief generated by the procedure can be transient because recurrent inflammation can cause new ductal injuries until the patient reaches adulthood, and the immune-mediated process regresses.^2^, ^16^, ^17^

The following sialoendoscopy findings were seen in the 174 procedures described in the medical records: stenosis, 44%; pallor, 18%; stones, 14%; and mucus plugs, 4%. The frequency of findings in the literature varies; however, these four described above are the most common.^9^

Sialoendoscopy-related complications are not common, especially when compared to complications related to the surgical excision of the glands, such as facial nerve, lingual, or hypoglossal nerves disfunction; intraoperative bleeding; postsurgical hematoma; surgical wound infection/abscess; salivary fistula; sialocele; Frey´s syndrome; and numbness in skin incision. In this study, complications of sialoendoscopy were (10.34%), transient, and similar to previous studies.^4^, ^18^, ^19^, ^20^ Among the complications, false path stands out and has been described as ductal perforation, false passage, false trajectory, irreparable damage to the ductal structure, ductal laceration, and papillary avulsion. False path is the exit of the endoscope from the duct path, which occurs most frequently in two locations: close to the ductal or intraductal ostium (when removing a stone or dilating a stenosis). It is not always possible to reinsert the scope to the duct after a false path, and in this study, false path occurred in eight of the 174 cases (4.59%).^4^, ^18^, ^19^, ^20^

Complications were approximately 3.43 times more likely to occur in the submandibular gland than in the parotid gland. The shorter anatomy of the duct and its curvature on the floor of the mouth make the procedure technically more challenge and more susceptible to false paths and restenosis. The sialolithiasis group also presented more complications than the other groups probably because combined approaches were used in >50% of cases. The incision made for combined access (both to catheterize the duct or remove the stone) can also occasionally damage neighboring structures, such as nerves, glandular tissues, and muscles, increasing the risk of complications.^21^, ^22^

Better success rates (91.2%) with fewer complications (7/114, 6.14%) were seen in case of procedures performed in the parotid gland ducts than in those performed in the submandibular gland ducts (86.7% and 11/60 [18.3%], respectively). A similar result was observed in a study on sialolithiasis conducted to investigate treatment outcomes between the glands. Of 155 cases of submandibular sialoendoscopy and 44 cases of sialoendoscopy in the parotid ducts, six submandibulectomies and one parotidectomy were scheduled due to procedure failure.^23^

Sialoendoscopy yielded positive results in several studies. In our study, the overall success rate was 90% (Fig. 1). Among the groups, the highest improvement rate was seen in the post-radioiodine therapy group (100%), and the least improvement, in the juvenile recurrent parotitis group (79%). However, this study did not discriminate between surgeries performed along the learning curve which could have affected the effectiveness rate.^24^

Several studies have confirmed that sialoendoscopy is both effective and safe. Consequently, knowledge regarding this procedure and its availability for the management of benign diseases of the salivary gland ducts have increased. Between 2006 and 2013, in the United States, studies involving 5,100 patients undergoing sialoendoscopy, sialoadenectomy, or both to treat salivary gland diseases evaluated the incidence of these procedures. The frequency of sialoendoscopies in this period increased from 0.13 to 0.42 per 100,000 inhabitants and of sialoadenectomies decreased from 2.41 to 1.43 per 100,000 inhabitants.^2^

## Conclusion

The majority of sialoendoscopies during the study period were performed in women and parotid gland ducts.

Complications were 3.43 times more likely to occur in procedures performed in the submandibular gland duct than in those performed in the parotid duct.

The sialolithiasis group was also more likely to experience recurrence and complications than the other groups.

Complications of sialoendoscopy were not unsignificant (10.34%) but transient in all. No serious or permanent surgical complications occurred.

The most frequent sialoendoscopy findings were stenosis (44%), pallor (18%), stones (20%), and mucus plugs (14%).

The overall success rate of sialoendoscopy was 90%. The most positive result was seen in the post-radioiodine therapy group (100%), and the least positive, in the juvenile recurrent parotitis group (79%).

## CRediT authorship contribution statement

Hannah Damasceno Barreto da Silva: Contributed to project design, literature review, data collection, writing, review and translation.

Carlos Takahiro Chone: Contributed to main guidance, project conception, writing and review.

José Higino Steck: Contributed to co-guidance, project conception, writing and review.

Samuel Serpa Steck: Contributed to data collection and writing.

Diogo Silva de Carvalho Guissoni: Contributed to data collection and writing.

## Declaration of competing interest

The authors declare no conflicts of interest.
